# Exercise ameliorates muscular excessive mitochondrial fission, insulin resistance and inflammation in diabetic rats via irisin/AMPK activation

**DOI:** 10.1038/s41598-024-61415-6

**Published:** 2024-05-09

**Authors:** Junjie Lin, Xin Zhang, Yu Sun, Haocheng Xu, Nan Li, Yuanxin Wang, Xin Tian, Chen Zhao, Bin Wang, Baishu Zhu, Renqing Zhao

**Affiliations:** College of Physical Education, Yangzhou University, Yangzhou, 225009 China

**Keywords:** Exercise training, Irisin, Mitochondrial fission, Inflammation, Diabetes, Endocrine system and metabolic diseases, Inflammation

## Abstract

This study aimed to investigate the effects of exercise on excessive mitochondrial fission, insulin resistance, and inflammation in the muscles of diabetic rats. The role of the irisin/AMPK pathway in regulating exercise effects was also determined. Thirty-two 8-week-old male Wistar rats were randomly divided into four groups (n = 8 per group): one control group (Con) and three experimental groups. Type 2 diabetes mellitus (T2DM) was induced in the experimental groups via a high-fat diet followed by a single intraperitoneal injection of streptozotocin (STZ) at a dosage of 30 mg/kg body weight. After T2DM induction, groups were assigned as sedentary (DM), subjected to 8 weeks of treadmill exercise training (Ex), or exercise training combined with 8-week cycloRGDyk treatment (ExRg). Upon completion of the last training session, all rats were euthanized and samples of fasting blood and soleus muscle were collected for analysis using ELISA, immunofluorescence, RT-qPCR, and Western blotting. Statistical differences between groups were analyzed using one-way ANOVA, and differences between two groups were assessed using t-tests. Our findings demonstrate that exercise training markedly ameliorated hyperglycaemia, hyperlipidaemia, and insulin resistance in diabetic rats (p < 0.05). It also mitigated the disarranged morphology and inflammation of skeletal muscle associated with T2DM (p < 0.05). Crucially, exercise training suppressed muscular excessive mitochondrial fission in the soleus muscle of diabetic rats (p < 0.05), and enhanced irisin and p-AMPK levels significantly (p < 0.05). However, exercise-induced irisin and p-AMPK expression were inhibited by cycloRGDyk treatment (p < 0.05). Furthermore, the administration of CycloRGDyk blocked the effects of exercise training in reducing excessive mitochondrial fission and inflammation in the soleus muscle of diabetic rats, as well as the positive effects of exercise training on improving hyperlipidemia and insulin sensitivity in diabetic rats (p < 0.05). These results indicate that regular exercise training effectively ameliorates insulin resistance and glucolipid metabolic dysfunction, and reduces inflammation in skeletal muscle. These benefits are partially mediated by reductions in mitochondrial fission through the irisin/AMPK signalling pathway.

## Introduction

Type 2 diabetes mellitus (T2DM) is a prevalent chronic condition that significantly impacts adult health globally^1–3^. The World Health Organization reports that in 2019, diabetes was responsible for 1.5 million deaths and there was a 3% increase in age-standardized death rates from diabetes between 2000 and 2019^4^. Insulin resistance (IR), commonly associated with T2DM, is often caused by mitochondrial dysfunction and chronic inflammation^5,6^. The skeletal muscle is the largest insulin-sensitive organ, which can absorb up to 80% of postprandial glucose and is crucial for regulating glucose and lipid metabolism. The function of mitochondria is central to the energy metabolism regulated by skeletal muscle. However, under the condition of T2DM, the homeostasis of mitochondria in skeletal muscle will be broken, and the biological properties of skeletal muscle will be impaired, which will affect the metabolism of the whole body^7,8^. Thus, understanding the mechanisms of mitochondrial homeostasis in skeletal muscle and developing effective interventions are imperative.

Mitochondria play a crucial role in energy metabolism^9^. Their biogenesis and quality control hinge on a dynamic balance between fusion and fission, which governs their morphology and distribution^10^. In nutrient-rich environments, mitochondria may fragment into numerous smaller vesicles with reduced functionality^11^. An increase in these vesicles beyond a certain threshold can trigger the release of reactive oxygen species and the activation of inflammatory pathways, contributing to insulin resistance^12,13^. A variety of proteins regulates the mitochondrial fission process. One such factor is Dynamin-related protein 1 (Drp1), which is recruited to the separation site of the mitochondrial outer membrane, where it combines with the fission protein 1 (Fis1) and the mitochondrial fission factor (MFF) to form a ring structure to contract the mitochondrial outer membrane resulting in the separation of mitochondria^14^. Research by Liu et al. indicated that in obese mice, elevated protein expression levels of Drp1 and Fis1 correlate with disrupted mitochondrial respiratory function^15^. Due to the essential role of mitochondrial homeostasis in maintaining cellular energy metabolism and preventing oxidative stress, which is related to the development of IR^16^, strategies that target boosting mitochondrial stability could be a viable strategy to decrease IR.

Regular exercise training is an effective strategy for reducing IR and preventing T2DM^17^. It enhances glucolipid metabolism and decreases inflammation, both of which contribute to mitigating IR^18,19^. Additionally, exercise reduces the expression of mitochondrial fission proteins, thereby curbing excessive mitochondrial fission. A recent study noted a marked reduction in Fis1 protein expression in the muscles of sedentary adults following aerobic exercise training, resulting in enhanced insulin sensitivity and lower fasting plasma glucose and fat mass^20^. However, the specific mechanisms through which exercise impacts excessive mitochondrial fission are not yet fully understood.

Increasing evidence indicates that exercise benefits health by stimulating the production of myokines, proteins released from muscles during exercise that target specific organs via the bloodstream^21–23^. One such myokine is irisin, derived from the cleavage of fibronectin type III domain-containing protein 5 (FNDC5) during exercise^24^. Bi et al. found that exogenous irisin treatment (250 μg/kg body weight) significantly diminished the expression of Drp1 and Fis1 in the liver of mice with hepatic ischemia–reperfusion injury, thereby inhibiting excessive mitochondrial fission^25^. Irisin also promotes energy metabolism, decreases inflammation, and alleviates IR, suggesting that it could modulate glucolipid metabolism and reduce inflammation by influencing mitochondrial fission^24,26,27^. Nevertheless, the specific mechanisms through which irisin affects mitochondrial fission factors require further investigation.

Irisin interacts with various myokines and factors to regulate energy metabolism during exercise. AMP-activated protein kinase (AMPK), a key regulator of exercise, maintains mitochondrial stability and manages metabolic processes in skeletal muscle^28^. Fan et al. have demonstrated that irisin can enhance AMPK phosphorylation and reduce the expression of Drp1 and MFF proteins in cardiomyocytes under high glucose conditions, this indicates that irisin collaborates with AMPK to maintain mitochondrial homeostasis^29^. Given the integral roles of irisin and AMPK in exercise physiology, we hypothesize that Irisin/AMPK could mitigate excessive mitochondrial fission in skeletal muscle during exercise, thus improving glucose and lipid metabolism, and reducing inflammation and IR.

## Materials and methods

### Experimental animal model and groups

The reporting of animal experiments follows the recommendations in the ARRIVE guidelines. Male Wistar rats (8-week-old) were purchased from the Comparative Medical Centre of Yangzhou University (Yangzhou, China). The rats were housed individually in separate cages subject to a 12-h light/dark cycle at a controlled temperature of 22–24 °C and relative humidity maintained at 50% ± 5%. They had ad libitum access to food and water. All experimental protocols followed the guidelines for using experimental animals and were approved by the Ethics Committee of Yangzhou University (No. 202303139).

**Figure 1:**
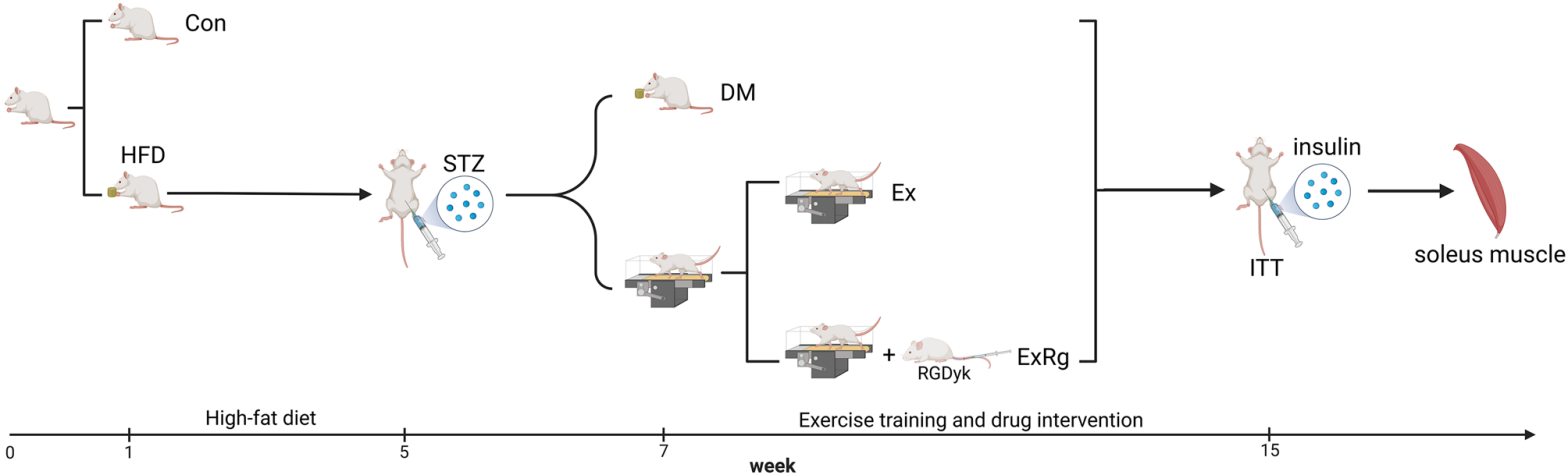
The schematic diagram of the experimental process. *Con* control, *DM* Type 2 diabetes, *Ex* Type 2 diabetes combined with exercise, *ExRg* Type 2 diabetes combined with exercise plus cycloRGDyk, *ITT* Insulin tolerance test, *HFD* High fat diet feeding.

All the rats were randomly divided into a control group (Con) and three experimental groups. The Con group was fed a standard diet (1010002, Jiangsu Xietong Medicine Bioengineering Co., China), while the experimental groups were fed a high-fat diet (XTHF45, Jiangsu Xietong Medicine Bioengineering Co., China). Four weeks later, rats in the experimental groups were given intraperitoneal injections of a single dose of streptozotocin (30 mg/kg, 18883-66-4, Sigma-Aldrich Co., USA)^30^. In contrast, the Con group received an equivalent dose of sodium citrate buffer via intraperitoneal injection. Seven days post-injection, rats exhibiting a fasting glucose level above 16.7 mmol/L were diagnosed with T2DM and subsequently divided into three subgroups: (1) a sedentary T2DM model group (DM); (2) an exercise training group (Ex), where DM rats underwent an 8-week exercise training intervention; (3) an exercise training and cycloRGDyk treatment group (ExRg), where DM rats received both eight weeks of exercise training and cycloRGDyk (irisin inhibitor) injection, with 8 rats in each group (Fig. 1)^31^.

### Exercise training and drug intervention protocols

Two weeks after streptozotocin injection, the rats in the Ex and ExRg groups commenced a treadmill exercise training regimen, conducted 5 days a week for 8 weeks. In the initial week, the rats underwent adaptive training; the treadmill speed was progressively increased from 10 to 18 m/min, the incline was set at a 5° uphill gradient, and the duration of exercise gradually extended from 10 to 45 min. From the second through the eighth week, each training session began with a 5-min warm-up at 10 m/min, after which the speed was raised to 18 m/min and maintained for 35 min before being reduced to 0 m/min. The exercise training protocol was designed as moderate-intensity treadmill training and was based on the internationally recognized Bedford exercise load standard. This regimen, including the duration and speed of exercise and the principle of progressive overload, was informed by the study conducted by Bedford et al.^32^.

During treadmill training, rats in the ExRg group were injected with cycloRGDyk (GC13923, GLPBIO Biological Company, USA) through the tail vein twice a week. CycloRGDyk was prepared in 10% DMSO (GK12002, GLPBIO Biological Company, USA) at a concentration of 1 mg/ml and administered at a dose of 2.5 mg/kg body weight^31,33^. Rats in the other groups received equivalent doses of 10% DMSO. The weight of the rats was measured prior to each injection to adjust the dosage accordingly.

### Insulin tolerance test (ITT)

Upon completion of the exercise training and pharmacological interventions, rats from each group were subjected to a 6-h fast. They then received an intraperitoneal injection of 0.5 IU/kg of regular human insulin. Blood glucose levels were measured prior to and at 30, 60, 90, and 120 min post-injection. Additionally, the area under the insulin tolerance curve was calculated to assess insulin sensitivity.

### Detection of glycolipid metabolism parameters

The fasting blood glucose (FBG) values of blood samples collected from the abdominal aorta of rats in each group were determined using a blood glucose detector. Subsequently, the remaining blood samples were centrifuged at 5000 rpm for 10 min to separate the serum for further biochemical analysis. Serum levels of total cholesterol (TC), triglyceride (TG), high-density lipoprotein cholesterol (HDL-C), low-density lipoprotein cholesterol (LDL-C) and glycated hemoglobin (HbA1c) were measured by an automatic biochemical analyser (BK-400, Biobase, China). Additionally, Serum irisin and fasting insulin (FIN) concentrations were assessed using enzyme-linked immunosorbent assay (ELISA) kits (JL21442-96T & JL10692-96T, Jianglai Biological Technology, China). The Homeostasis Model Assessment of Insulin Resistance (HOMA-IR) was calculated using the formula: HOMA-IR = FBG (mmol/L) × FIN (mU/L)/22.5^34^.

### H&E staining

Fresh soleus muscle from the left side of the rat was excised and fixed in a 4% paraformaldehyde solution to preserve its structural integrity. After fixation, the soleus muscle samples were dehydrated through a graded series of ethanol concentrations. The dehydrated samples were subsequently processed with xylene and embedded in paraffin for sectioning. Following embedding and sectioning, 5 µm thick paraffin sections of soleus muscle tissue were prepared^35^.

The paraffin was then removed from the sections using xylene to prepare them for staining. Initially, nuclei were selectively stained blue-purple with hematoxylin. Following rinsing, cytoplasmic components and extracellular matrix elements were stained pink with eosin, creating a stark contrast with the hematoxylin-stained nuclei^35^. The stained sections were then dehydrated, cleared, and sealed with resin before being examined under an optical microscope (Nikon, Japan).

### Immunofluorescence analysis

The sections cut from soleus muscle tissue were deparaffinized and subjected to antigen retrieval under high temperature and pressure. The sections were then blocked with 5% skim milk powder for two hours and subsequently incubated with primary antibodies against IL-1β (1:250, SC-12742, Santa Cruz), TNF-α (1:250, SC-52746, Santa Cruz), Drp1 (1:200, A17069, ABclonal), and Fis1 (1:200, A19666, ABclonal) overnight at 4 °C. After rinsing off the primary antibodies, the sections were incubated with the corresponding secondary antibodies for 2 h. The target proteins in the soleus muscle sections were then visualized using fluorescently labeled tyramine (G1222, Servicebio, China), washed with TBST, and counterstained with DAPI (G1012, Servicebio, China). Images were acquired with a confocal microscope (Carl Zeiss AG, Germany) and analyzed quantitatively using Image-J software.

### Real-time quantitative PCR analysis

Total RNA extracted from the right soleus muscle of each group of rats by using Trizol reagent and an equal amount of RNA was reverse transcribed into cDNA using HiScript II Q RT SuperMix for qPCR (R223-01, Vazyme, China). For quantitative PCR, the reagents were added sequentially to a 96-well optical reaction plate and then centrifuged briefly at 2000 rpm to ensure that the mixture was fully settled at the bottom of the wells. The reflector plate was placed in the Real Time PCR System, and amplification was conducted following the protocol of the qPCR SYBR Green Master Mix (Q121-02, Vazyme, China). The primers, synthesized by GENERAL BIOL, are listed in Table 1. To determine primer efficiency, a series of tenfold dilutions of cDNA were prepared, and repeated qPCR analyses were conducted to establish a standard curve. Primer efficiency (E) is calculated using the slope value with the following formula: E = 10^−1/slope^ − 1. Subsequently, the expression of the target gene in the rat soleus muscle was quantified using the comparative method (2^−△△Ct^), with GAPDH serving as the reference gene.

**Table 1:** Primer sequence for real-time qPCR.

Gene	Sense (5′–3′)	Antisense (5′–3′)	E
Drp1	CAGCGAGATTGTGAGGTTATTGA	CGGATTCAGTCAGAAGGTCATC	94.6%
Fis1	ATCCGTAGAGGCATCGTG	CCTTGAGCCGGTAGTTG	96.3%
MFF	CTGCCGCCACTTCTAATCCTCATC	TCAATGAAGCCGCATCTACCACAG	95.9%
MFN2	CCATGATGCCCAACCTGTGA	TCCTGTGGGTGTGTCTTCAAGGA	94.7%
FNDC5	AAGTGGTCATTGGCTTTGC	GTTGTTATTGGGCTCGTTGT	92.8%
GAPDH	TGCTTCACCACCTTCTTGA	TCACCATCTTCCAGGAGC	99.2%

### Western blot analysis

Protein concentrations in 30 mg of soleus muscle tissue extracted from the right limb of rats were measured using a BCA kit. Subsequently, 45 µg of protein, dissolved in loading buffer, was subjected to electrophoresis and then transferred onto polyvinylidene difluoride (PVDF) membranes. After blocking with 5% skim milk powder, the PVDF membranes were incubated with primary antibodies against IL-1β (1:1000, SC-12742, Santa Cruz), TNF-α (1:1000, SC-52746, Santa Cruz), Drp1 (1:2000, A17069, ABclonal), Fis1 (1:2000, A19666, ABclonal), MFF (1:2000, GB114102, Servicebio), MFN2 (1:5000, 12186-1-AP, Proteintech), FNDC5 (1:2000, A18107, ABclonal), AMPK (1:500, A12718, ABclonal), and p-AMPK (1:1000, AP0432, ABclonal) for 12 h at 4 °C. This was followed by incubation with the appropriate secondary antibodies. The expression of the target proteins was analyzed using ImageJ software, with β-actin (1:50,000, AC048, ABclonal) serving as a reference.

### Statistical analysis

For statistical analysis of the experimental data, we utilized GraphPad Prism software (version 9.5.0, CA, USA). Results are presented as mean ± standard deviation (SD). To determine statistical differences among multiple groups, one-way analysis of variance (ANOVA) was employed, with post-hoc comparisons conducted using Tukey's or Bonferroni correction method to control for multiple testing errors. Data normality was assessed using the Shapiro–Wilk test prior to applying ANOVA. For comparing two independent groups, an unpaired two-tailed t-test was used. All tests were two-sided, and a p-value of less than 0.05 was considered statistically significant.

## Results

### Exercise ameliorates hyperglycaemia, hyperlipidaemia, and insulin resistance in diabetic rats

**Figure 2:**
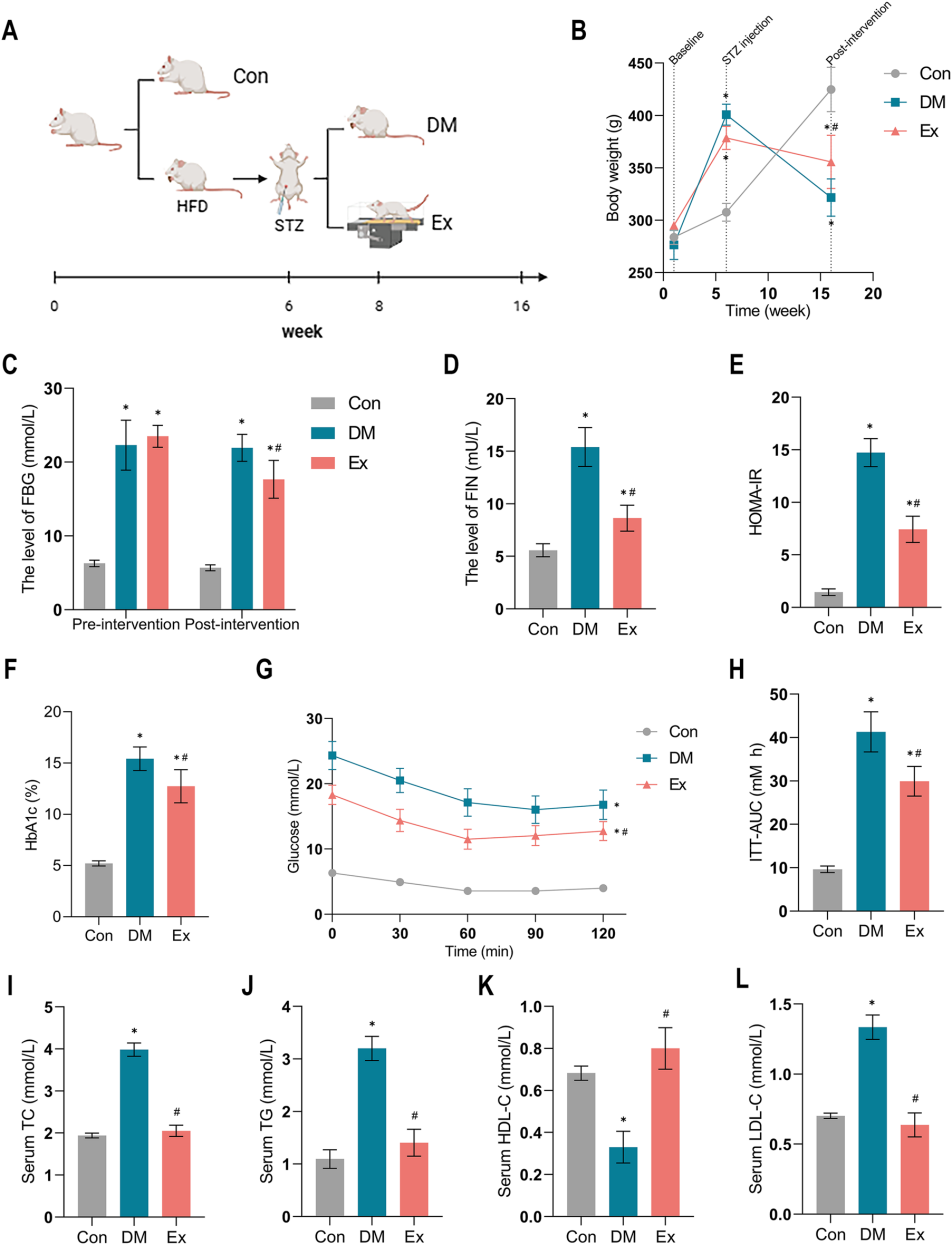
Physiological parameters of rats between groups. (**A**) Experimental schedules. (**B**) Trends in body weight. (**C**,**D**) Levels of FBG and FIN. (**E**) Insulin resistance index. (**F**) Levels of HbA1c. (**G**) Fasting blood glucose level was determined after insulin injection for 30, 60, 90 and 120 min. (**H**) Area under the insulin tolerance curve in different groups. (**I**–**L**) Levels of lipid parameters. *Con* control, *DM* Type 2 diabetes, *Ex* Type 2 diabetes combined with exercise, *TC* Total cholesterol, *TG* Triglyceride, *HDL-C* High-density lipoprotein cholesterol, *LDL-C* low-density lipoprotein cholesterol, *FBG* Fasting blood glucose, *FIN* Fasting insulin, *HbA1c* Glycated haemoglobin, *ITT* Insulin tolerance test. *Denotes the significant difference compared with Con group (p < 0.05); ^**#**^indicates the significant difference compared with DM group (p < 0.05).

We established a diabetic rat model using a combined protocol of high-fat diet feeding and STZ injection, as previously described^30^. Following T2DM induction, the rats consistently exhibited hyperglycemia (> 16.7 mmol/L) over three consecutive days, confirming the successful establishment of the T2DM model^30^. At the end of the 8-week intervention protocol, DM rats demonstrated a significant reduction in body weight, whereas control group rats gained weight relative to their initial measurements at the study's onset. Notably, the body weight of DM rats receiving exercise training slightly decreased after the 8-week intervention, but it was better than sedentary DM rats (p < 0.05). These results underscore the detrimental effects of T2DM on body weight and suggest the potential for exercise training to mitigate these effects (Fig. 2).

In the absence of any intervention, the diabetic rats experienced elevated levels of HbA1c, FBG, FIN, and significant insulin resistance (measured by HOMA-IR and ITT) compared to normal rats (p < 0.05). However, these adverse effects, including hyperglycemia and insulin resistance, were ameliorated following 8 weeks of treadmill exercise training (p < 0.05). The same period of exercise training also significantly mitigated hyperlipidemia. These findings highlight the efficacy of exercise training as a therapeutic strategy to manage conditions induced by T2DM, such as hyperglycemia, hyperlipidemia, and insulin resistance (Fig. 2).

### Exercise improves morphology and inflammation of skeletal muscle in diabetic rats

**Figure 3:**
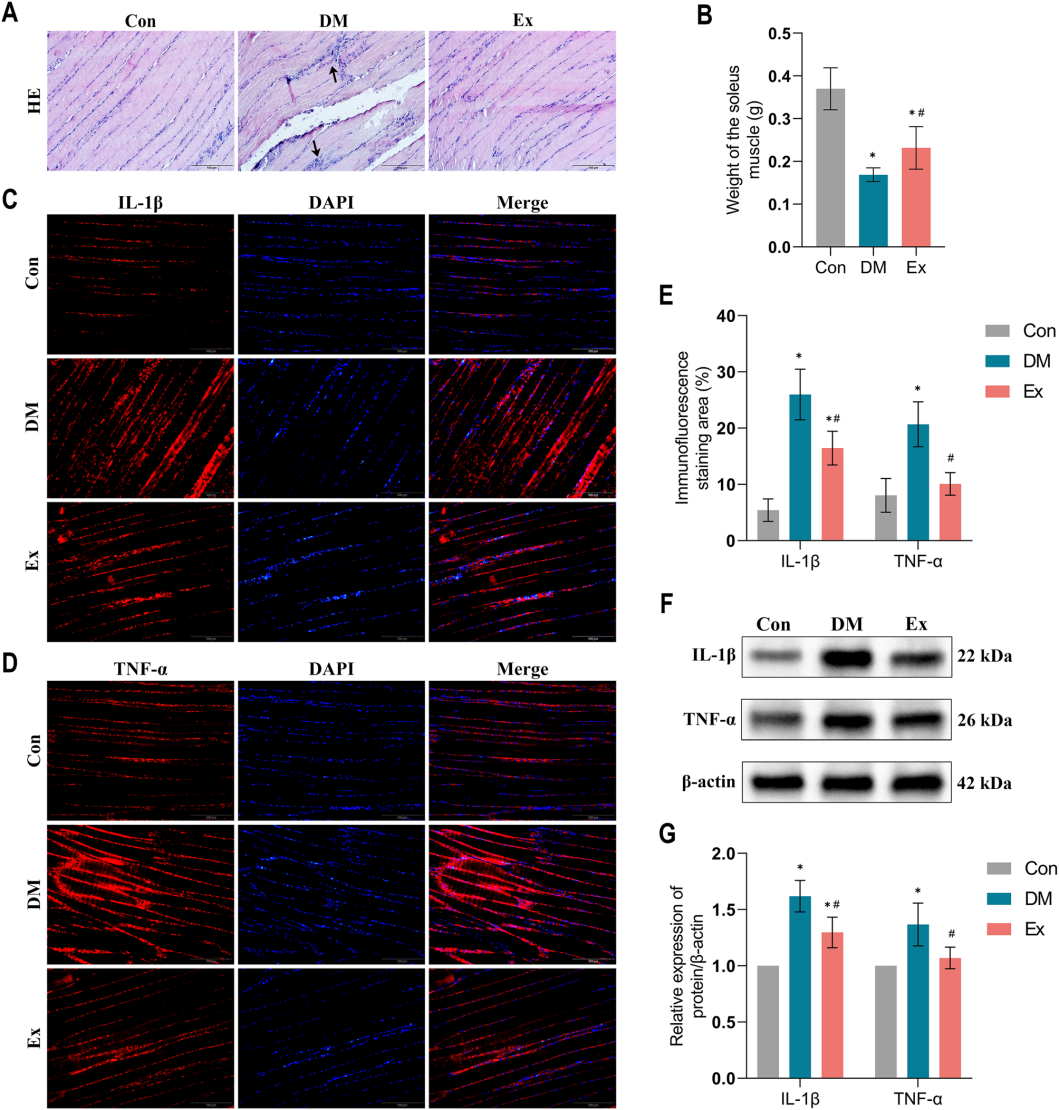
Exercise improves morphology and inflammation of skeletal muscle in diabetic rats. (**A**) H&E staining of representative Skeletal muscle sections. (**B**) Weight of soleus muscle. (**C**–**E**) Immunofluorescence and its semiquantitative analysis for evaluating IL-1β and TNF-α expression in soleus muscle of rats between groups. (**F**–**G**) Western blot analysis of IL-1β and TNF-α expression in soleus muscle of rats between groups. *Con* control, *DM* Type 2 diabetes, *Ex* Type 2 diabetes combined with exercise, *IL-1β* interleukin-1β, *TNF-α* tumor necrosis factor-α. *Denotes the significant difference compared with Con group (p < 0.05); ^**#**^indicates the significant difference compared with DM group (p < 0.05).

Skeletal muscle plays a vital role in glucose transport and is a primary site of IR in T2DM^8^. Previous research has shown that skeletal muscle dysfunction and chronic inflammation are pivotal in T2DM pathology^36^. To assess the effects of T2DM on soleus muscle morphology and inflammation, we measured the weight and histological features of the soleus muscle in the left hind limb of DM rats. The soleus muscle weight in the DM group was significantly reduced compared to the control group (Fig. 3). Notably, eight weeks of treadmill exercise training intervention substantially reversed the T2DM-related weight loss in these muscles (p < 0.05). Moreover, while muscle fibers in the control group were regularly aligned, those in the DM rats were disorganized and fragmented, with considerable gaps filled with inflammatory cells. This structural damage was notably remedied following the exercise training intervention, alongside a reduction in inflammatory cell presence. Further, we utilized immunofluorescence and Western blot analyses to quantify the inflammatory markers IL-1β and TNF-α in the rat soleus muscle. These markers were significantly elevated in the DM group but were markedly reduced after the exercise training intervention (p < 0.05). Collectively, these findings suggest that treadmill exercise training can significantly restore skeletal muscle integrity and diminish inflammation in diabetic conditions.

### Exercise suppresses muscular excessive mitochondrial fission in diabetic rats

Increasing evidence suggests a correlation between IR, chronic inflammation, and mitochondrial dysfunction, including disruptions in mitochondrial fusion and fission processes^15,37^. We investigated the impact of T2DM on the mitochondrial quality control system through immunofluorescence analyses, protein synthesis evaluations, and transcriptional studies.

**Figure 4:**
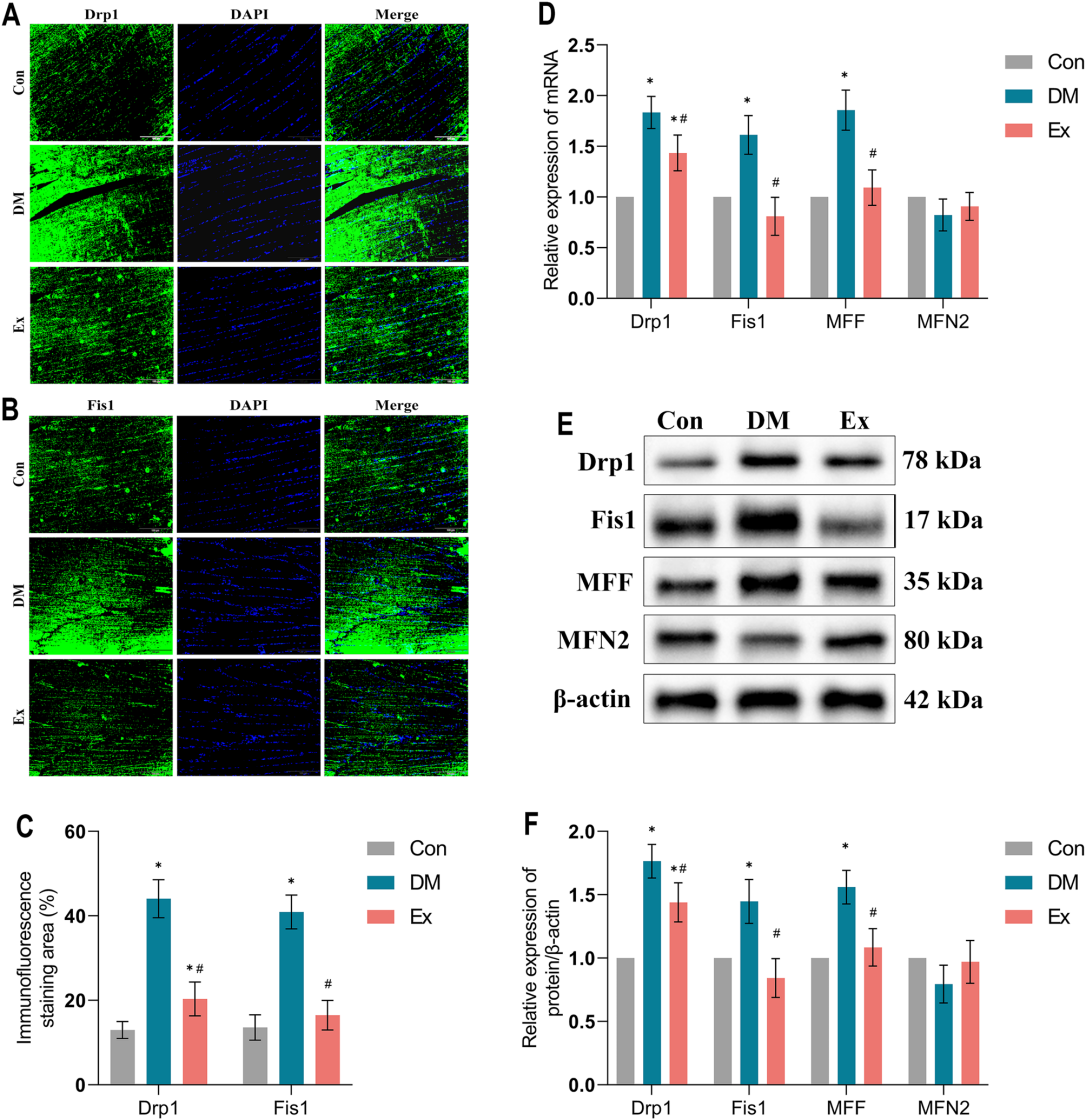
Exercise suppresses muscular excessive mitochondrial fission in diabetic rats. (**A**–**C**) Immunofluorescence and its semiquantitative analysis for evaluating Drp1 and Fis1 expression in soleus muscle of rats between groups. (**D**) RT-qPCR analysis of the mRNA expression of Drp1, Fis1, MFF and MFN2 in soleus muscle of rats between groups. (**E**,**F**) Western blot analysis of Drp1, Fis1, MFF and MFN2 expression in soleus muscle of rats between groups. *Con* control, *DM* Type 2 diabetes, *Ex* Type 2 diabetes combined with exercise, *Drp1* Dynamin-related protein 1, *Fis1* Fission protein 1, *MFF* Mitochondrial fission factor, *MFN2* Mitofusin 2. *Denotes the significant difference compared with Con group (p < 0.05); ^**#**^indicates the significant difference compared with DM group (p < 0.05).

In DM group, the fluorescence area of mitochondrial fission proteins Drp1 and Fis1 was significantly higher than in the control group, indicating enhanced mitochondrial fission in skeletal muscle (Fig. 4). This aberrant fission was substantially mitigated after an 8-week treadmill exercise training regimen in the Ex group, where Drp1 and Fis1 fluorescence area markedly decreased (p < 0.05). Additionally, mRNA and protein levels of Drp1, Fis1, and MFF were considerably elevated in the soleus muscle of DM rats relative to the Con group. Post-exercise training intervention, these levels significantly declined (p < 0.05). Conversely, the expression of the mitochondrial fusion protein MFN2 decreased in DM rats and partially recovered following exercise training, although these changes were not statistically significant. These results demonstrate that exercise training can effectively reduce excessive mitochondrial fission in skeletal muscle of diabetic rats.

### Exercise up-regulated irisin/AMPK expression, but this is inhibited by cycloRGDyk treatment

**Figure 5:**
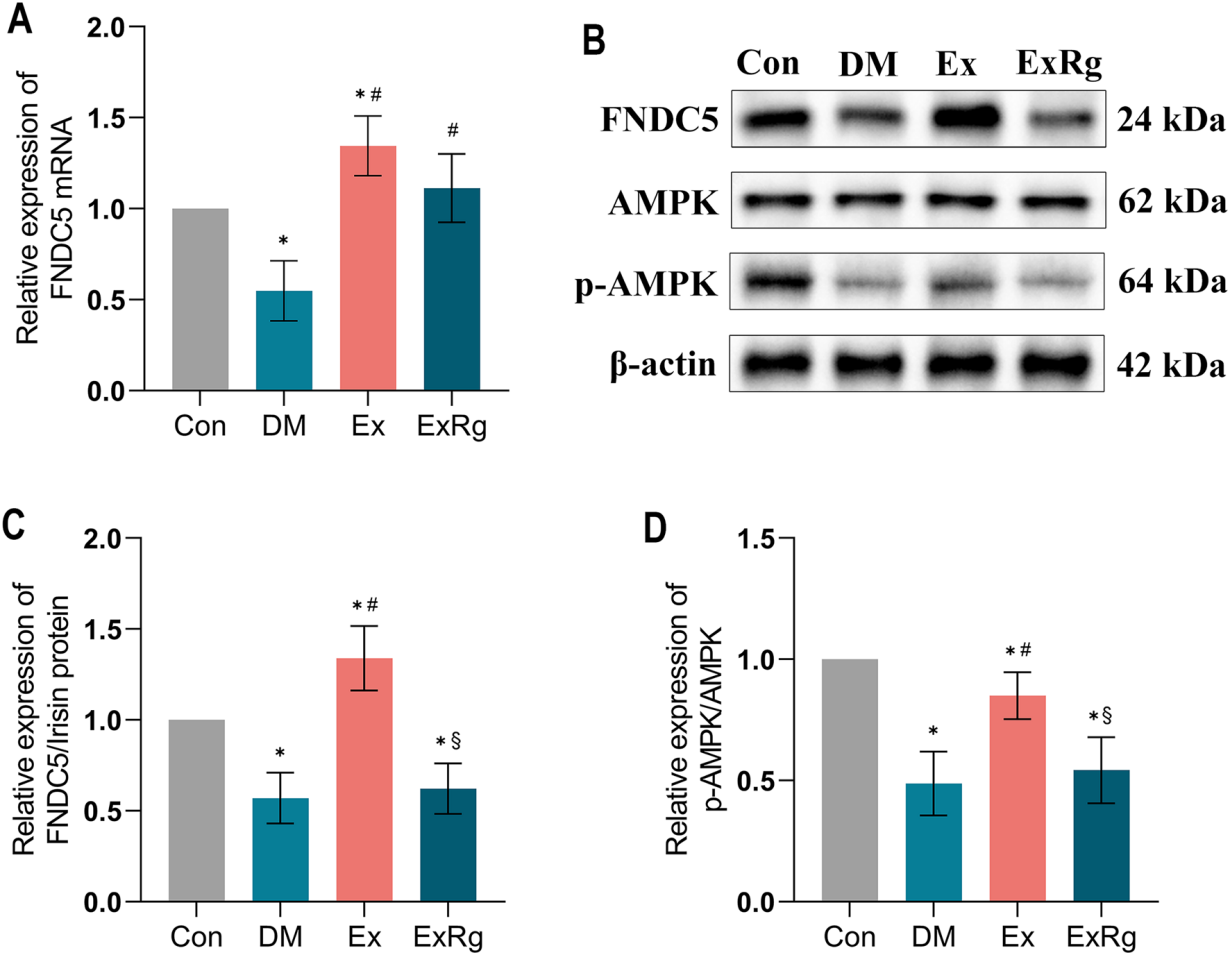
Exercise and cycloRGDyk treatment affect the Irisin/AMPK pathway in the skeletal muscle of diabetic rats. (**A**) RT-qPCR analysis of soleus muscle FNDC5 expressions. (**B**–**D**) Western blot analysis of FNDC5/irisin and p-AMPK expression in soleus muscle of rats between groups. *Con* control, *DM* Type 2 diabetes, *Ex* Type 2 diabetes combined with exercise, *ExRg* Type 2 diabetes combined with exercise plus cycloRGDyk, *FNDC5* Fibronectin type III domain-containing protein 5, *AMPK* AMP-activated protein kinase. *Denotes the significant difference compared with Con group (p < 0.05); ^**#**^indicates the significant difference compared with DM group (p < 0.05); ^**§**^presents the significant difference compared with Ex group (p < 0.05).

Irisin, a myokine discovered recently, is recognized for linking exercise with metabolic disorders^24,26^. Our prior research demonstrated that irisin secretion induced by exercise plays a crucial role in regulating glucose transport, lipid metabolism, and IR^38^. These findings suggest that irisin may be the pivotal mediator of exercise's beneficial effects on energy metabolism. To explore the influence of treadmill exercise training on irisin levels, we analyzed its expression in the soleus muscle. We found that both FNDC5 mRNA and the FNDC5/irisin protein levels were significantly reduced in DM rats compared to controls (Fig. 5). However, these levels substantially recovered after an 8-week exercise training regimen (p < 0.05). Further studies have indicated that irisin often interacts with other molecular signals to enhance exercise's positive impacts on metabolic diseases, such as through the phosphorylation of AMPK, another key myokine involved in metabolic processes^39^. Consequently, we investigated whether blocking irisin signaling would alter exercise-regulated AMPK phosphorylation. We observed that phosphorylation levels of AMPK were lower in diabetic rats than in controls, but 8 weeks of exercise training significantly reversed this reduction (p < 0.05). Nevertheless, the enhanced phosphorylation of AMPK due to exercise training was inhibited by blocking irisin receptor signaling. This indicates that the beneficial effects of exercise training on diabetic rats might be partly due to the upregulation of irisin, which facilitates AMPK phosphorylation.

### Exercise-related beneficial effects could be partially blocked by cycloRGDyk treatment in diabetic rats

**Figure 6:**
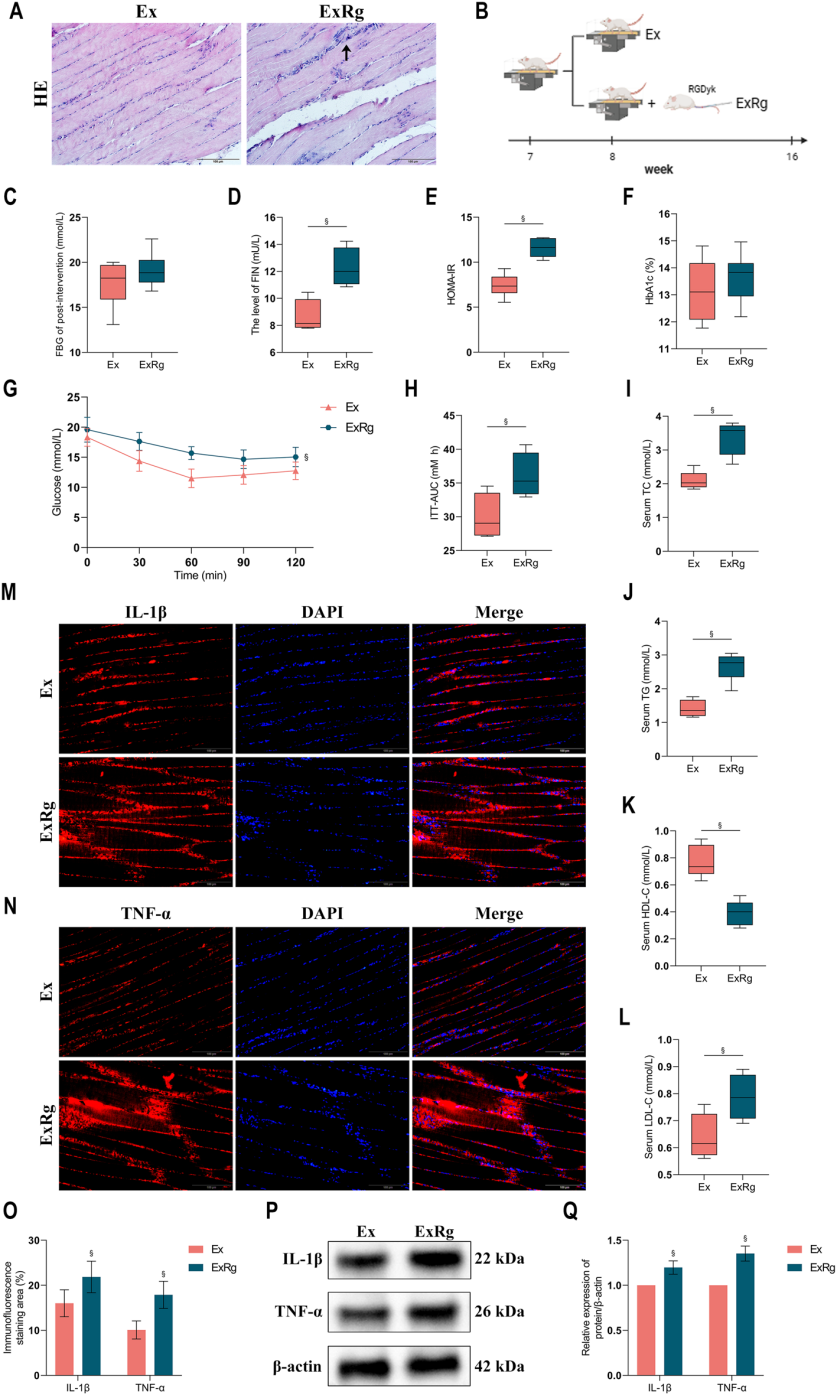
Exercise-related beneficial effects could be partially blocked by cycloRGDyk treatment in diabetic rats. (**A**) H&E staining of representative Skeletal muscle sections. (**B**) Experimental schedules. (**C**,**D**) Levels of FBG and FIN. (**E**) Insulin resistance index. (**F**) Levels of HbA1c. (**G**) Fasting blood glucose level was determined after insulin injection for 30, 60, 90 and 120 min. (**H**) Area under the insulin tolerance curve in different groups. (**I–L**) Levels of lipid parameters. (**M–O**) Immunofluorescence and its semiquantitative analysis for evaluating IL-1β and TNF-α expression in soleus muscle of rats between groups. (**P**–**Q**) Western blot analysis of IL-1β and TNF-α expression in soleus muscle of rats between groups. *Ex* Type 2 diabetes combined with exercise, *ExRg* Type 2 diabetes combined with exercise plus cycloRGDyk, *FBG* Fasting blood glucose, *FIN* Fasting insulin, *TC* Total cholesterol, *TG* Triglyceride, *HDL-C* High-density lipoprotein cholesterol, *LDL-C* Low-Density Lipoprotein Cholesterol, *IL-1β* interleukin-1β, *TNF-α* tumor necrosis factor-α, *HbA1c* Glycated haemoglobin, *ITT* Insulin tolerance test. ^§^Presents the significant difference compared with the Ex group (p < 0.05).

We investigated whether inhibiting irisin signaling could obstruct the positive effects of exercise training on hyperglycemia, hyperlipidemia, IR, and inflammation in diabetic conditions. H&E staining revealed that blocking the irisin receptor pathway diminished the beneficial enhancements in skeletal muscle fiber characteristics typically induced by exercise training. This led to greater disorganization of muscle fibers, and enhanced infiltration of inflammatory cells. Administration of cycloRGDyk similarly curtailed improvements in FIN levels, HOMA-IR scores, and insulin tolerance associated with exercise training (Fig. 6). Furthermore, cycloRGDyk treatment effectively obstructed positive alterations in hyperlipidemia observed in diabetic rats undergoing exercise training (p < 0.05). Additionally, cycloRGDyk reduced the anti-inflammatory effects of exercise training, as indicated by increased protein expression and fluorescence imaging of IL-1β and TNF-α in the soleus muscle of exercised rats receiving this treatment (p < 0.05).

### The effect of exercise in improving muscular excessive mitochondrial fission was blocked by cycloRGDyk injection

**Figure 7:**
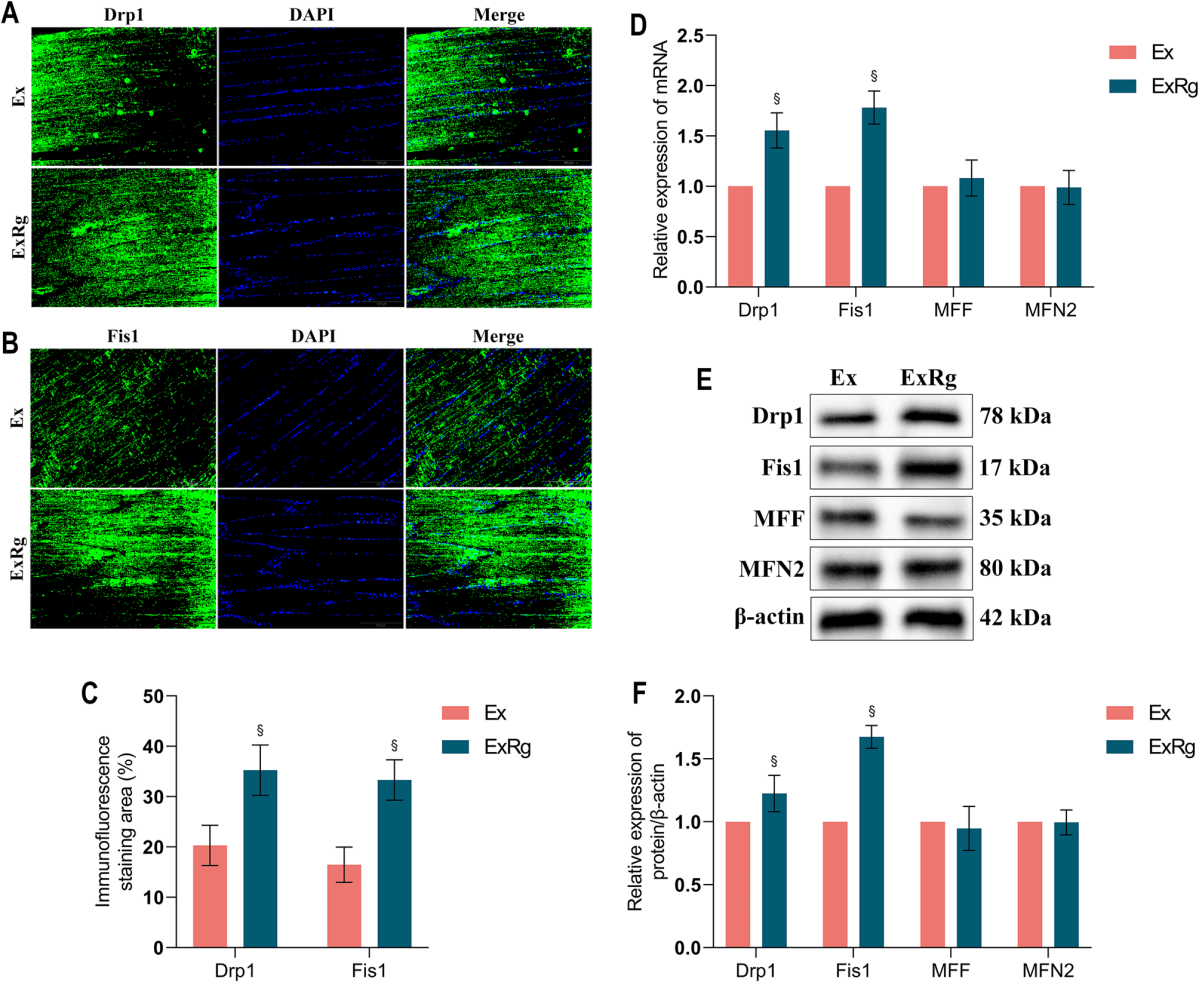
The effect of exercise in regulating muscular excessive mitochondrial fission was blocked by cycloRGDyk injection. (**A**–**C**) Immunofluorescence and its semiquantitative analysis for evaluating Drp1 and Fis1 expression in soleus muscle of rats between groups. (**D**) RT-qPCR analysis of the mRNA expression of Drp1, Fis1, MFF and MFN2 in soleus muscle of rats between groups. (**E**,**F**) Western blot analysis of Drp1, Fis1, MFF and MFN2 expression in soleus muscle of rats in Ex and ExRg group. *Ex* Type 2 diabetes combined with exercise, *ExRg* Type 2 diabetes combined with exercise plus cycloRGDyk, *Drp1* Dynamin-related protein 1, *Fis1* Fission protein 1, *MFF* Mitochondrial fission factor, *MFN2* Mitofusin 2. ^§^Presents the significant difference compared with the Ex group (p < 0.05).

To explore the role of irisin signaling in mitigating excessive mitochondrial fission induced by exercise training, diabetic rats undergoing treadmill routines were treated with cycloRGDyk. Immunofluorescence analysis indicated that the fluorescence intensities of Drp1 and Fis1 proteins were significantly elevated in the ExRg group compared to the exercise training-only group (Ex). Subsequent Western blot and RT-qPCR analyses showed that the expressions of mitochondrial fission proteins Drp1 and Fis1 in the ExRg group were markedly higher than those in the Ex group (p < 0.05), as depicted in Fig. 7. These results underscore the importance of irisin signaling in controlling mitochondrial fission in the skeletal muscle of diabetic rats engaged in treadmill exercise training.

## Discussion

Our findings indicate that exercise training activates the irisin/AMPK pathway, thereby decreasing excessive mitochondrial fission in skeletal muscle. This process is linked to the amelioration of glucolipid metabolism and the reduction of IR and inflammation associated with T2DM, aligning with our initial hypothesis. While previous research has explored the interaction between irisin and mitochondrial dynamics during exercise in various disease models, studies involving T2DM rodent models have been lacking^40^. This study contributes novel experimental evidence that addresses this gap in the literature.

Extensive research has established that regular exercise offers multiple benefits for T2DM management, including enhanced glucose uptake, increased muscle mass, accelerated lipid metabolism, and improved insulin sensitivity^41–43^. Our results are consistent with these findings. However, these positive effects were attenuated by the administration of an irisin antagonist (RGDyk), indicating the potential therapeutic role of irisin in T2DM management. Zheng et al. demonstrated that irisin significantly boosts the PI3K/AKT insulin signaling pathway both in vivo and in vitro, and markedly reduces serine phosphorylation of IRS-1 and IRS-2, which aligns with our observations that irisin enhances insulin sensitivity in diabetic rats^39^. In this study, we monitored the dynamic insulin response in rats via insulin tolerance tests and computed the HOMA-IR index as a static measure of insulin resistance. The integration of these dynamic and static metrics confirmed that irisin contributes to improved insulin sensitivity and reduced insulin resistance during exercise training. Moreover, Luo et al. reported that mice deficient in FNDC5/irisin displayed lower HDL-C levels, higher LDL-C levels, and significantly decreased insulin sensitivity, findings that resonate with our own^44^. Additional research has elucidated the anti-inflammatory effects and mechanisms of irisin across various animal and cell models^27,39,45^, suggesting that irisin signaling enhances the beneficial impacts of exercise on T2DM.

The beneficial impact of exercise on T2DM management is closely associated with improved mitochondrial homeostasis. Studies have established that mitochondrial fission is vital for maintaining mitochondrial integrity in skeletal muscle cells, which is crucial for optimal energy metabolism^15,37,46^. Our findings indicate that diabetic rats exhibited elevated levels of mitochondrial fission regulatory proteins such as Drp1, Fis1, and MFF, correlated with increased mitochondrial fission, an abundance of damaged mitochondria, and heightened inflammatory pathways. Regular physical activity has been demonstrated to facilitate the removal of damaged or dysfunctional mitochondria and to stimulate mitochondrial biogenesis—the formation of new mitochondria^47^. This process leads to an increase in the number of functional mitochondria in skeletal muscle cells, thereby boosting energy production and enhancing metabolic health. Research by Fealy et al. showed that 12 weeks of aerobic exercise decreased Drp1 activity in the skeletal muscles of obese individuals, improving their insulin sensitivity and fat oxidation^48^. Likewise, our results imply that exercise training may enhance glycolipid metabolism by reducing excessive mitochondrial fission in skeletal muscle.

Irisin, a myokine produced through the activation of PGC-1α (Peroxisome Proliferator-Activated Receptor Gamma Coactivator 1-Alpha) during exercise^24^, plays a crucial role in cellular energy metabolism. PGC-1α activates transcription factors that enhance gene expression for mitochondrial complexes, thus improving mitochondrial function. This enhancement is potentially linked to irisin^49^. Zhang et al. discovered that exogenous irisin can enhance the activity of mitochondrial complex I in the brains of mice with Parkinson's disease, thereby improving mitochondrial function^50^. Similarly, Bi et al. demonstrated that irisin administration significantly alleviated the expression of Drp1 and Fis1 in the liver of mice with hepatic injury, inhibiting excessive mitochondrial fission^25^. This finding parallels those of the current study. In this study, we chose the soleus muscle for analysis to evaluate the role of irisin in skeletal muscle. Soleus muscle is one of the major skeletal muscles stimulated by mechanical forces during treadmill training, and can secrete large amounts of irisin. In addition, Chen et al. reported that oxidative muscles such as soleus muscle can secrete more irisin than glycolytic muscles such as gastrocnemius or quadriceps muscle^51^. The results we obtained suggest that irisin, which binds to the αVβ5 integrin, may regulate excessive mitochondrial fission in the skeletal muscle of diabetic rats during exercise.

Extensive research has shown that irisin collaborates with various myokines and metabolic factors to regulate energy metabolism during exercise^39,52^. Specifically, AMPK serves as a crucial downstream signaling molecule for irisin, essential for maintaining mitochondrial homeostasis and managing glucose and lipid metabolism^28^. Our research indicates that AMPK activation is closely linked to exercise training, as evidenced by enhanced phosphorylation at Thr172 in T2DM rats during treadmill running, an effect that is inhibited by an irisin antagonist (RGDyk). Studies by Tang et al. and Lee et al. have further established that irisin treatment augments AMPK phosphorylation at Thr172 in a time- and dose-dependent manner^53,54^. These findings highlight the role of irisin in promoting AMPK phosphorylation during exercise. Moreover, it has been demonstrated that AMPK activation facilitates AKAP1 phosphorylation at Ser103 and its interaction with protein kinase A (PKA), which in turn reduces the activity and expression of Drp1^48,55,56^. Consequently, the irisin/AMPK pathway may mitigate excessive mitochondrial fission in the skeletal muscle of T2DM rats during exercise.

In summary, our research supports the hypothesis that exercise training can enhance glycolipid metabolism, insulin resistance (IR), and inflammation by reducing excessive mitochondrial fission in the skeletal muscle of diabetic rats. Our findings also suggest that the irisin/AMPK pathway plays a role in this process. However, our study has several limitations that future research should address. Primarily, while this study provides in vivo evidence of the effects of exercise on mitochondrial dysfunction and IR in diabetic rats, including the role of the irisin/AMPK pathway, further in vitro studies are necessary to clarify how irisin influences the molecular mechanisms involved, potentially through cell culture and transgenic experiments in diabetic models. Moreover, while we have shown that the irisin/AMPK pathway can decrease insulin resistance and improve insulin sensitivity during exercise, its specific impacts on insulin resistance pathways remain unclear. Additionally, our study does not include measurements of mitochondrial function markers such as mitochondrial complexes. Furthermore, the lack of an Oral Glucose Tolerance Test (OGTT) also constitutes a limitation. Consequently, more studies are essential to explore these aspects thoroughly.

### Supplementary Information

Supplementary Figures.

## Data Availability

The authors of this article will make the raw data supporting their conclusions available, without any hesitation or reservation. Renqing Zhao (renqing.zhao@yzu.edu.cn) should be contacted if someone wants to request the data from this study.

## References

[1] Lean, M. E. J. *et al.* Durability of a primary care-led weight-management intervention for remission of type 2 diabetes: 2-year results of the DiRECT open-label, cluster-randomised trial. *Lancet Diabetes Endocrinol.* **7**(5), 344–355. 10.1016/S2213-8587(19)30068-3 (2019). 30852132 10.1016/S2213-8587(19)30068-3

[2] Collaborators, G. B. D. D. Global, regional, and national burden of diabetes from 1990 to 2021, with projections of prevalence to 2050: A systematic analysis for the Global Burden of Disease Study 2021. *Lancet* **402**(10397), 203–234. 10.1016/S0140-6736(23)01301-6 (2023). 37356446 10.1016/S0140-6736(23)01301-6 PMC10364581

[3] Bellary, S. *et al.* Type 2 diabetes mellitus in older adults: Clinical considerations and management. *Nat. Rev. Endocrinol.* **17**(9), 534–548. 10.1038/s41574-021-00512-2 (2021). 34172940 10.1038/s41574-021-00512-2

[4] World Health Organization. Diabetes. https://www.who.int/news-room/fact-sheets/detail/diabetes (accessed 23 Oct 2023).

[5] Esser, N. *et al.* Inflammation as a link between obesity, metabolic syndrome and type 2 diabetes. *Diabetes Res. Clin. Pract.* **105**(2), 141–150. 10.1016/j.diabres.2014.04.006 (2014). 24798950 10.1016/j.diabres.2014.04.006

[6] Agbu, P. & Carthew, R. W. MicroRNA-mediated regulation of glucose and lipid metabolism. *Nat. Rev. Mol. Cell Biol.* **22**(6), 425–438. 10.1038/s41580-021-00354-w (2021). 33772227 10.1038/s41580-021-00354-w PMC8853826

[7] Huh, J. Y. *et al.* FNDC5 and irisin in humans: I. Predictors of circulating concentrations in serum and plasma and II. mRNA expression and circulating concentrations in response to weight loss and exercise. *Metabolism* **61**(12), 1725–1738. 10.1016/j.metabol.2012.09.002 (2012). 23018146 10.1016/j.metabol.2012.09.002 PMC3614417

[8] Chen, Q. *et al.* Targeting RalGAPalpha1 in skeletal muscle to simultaneously improve postprandial glucose and lipid control. *Sci. Adv.* **5**(4), eaav4116. 10.1126/sciadv.aav4116 (2019). 30989113 10.1126/sciadv.aav4116 PMC6459767

[9] Yan, W., Diao, S. & Fan, Z. The role and mechanism of mitochondrial functions and energy metabolism in the function regulation of the mesenchymal stem cells. *Stem Cell Res. Ther.* **12**(1), 140. 10.1186/s13287-021-02194-z (2021). 33597020 10.1186/s13287-021-02194-z PMC7890860

[10] Sabouny, R. & Shutt, T. E. Reciprocal regulation of mitochondrial fission and fusion. *Trends Biochem. Sci.* **45**(7), 564–577. 10.1016/j.tibs.2020.03.009 (2020). 32291139 10.1016/j.tibs.2020.03.009

[11] Zorov, D. B. *et al.* Lessons from the discovery of mitochondrial fragmentation (fission): A review and update. *Cells*10.3390/cells8020175 (2019). 30791381 10.3390/cells8020175 PMC6406845

[12] Harrington, J. S. *et al.* Mitochondria in health, disease, and aging. *Physiol. Rev.* **103**(4), 2349–2422. 10.1152/physrev.00058.2021 (2023). 37021870 10.1152/physrev.00058.2021 PMC10393386

[13] Samuel, V. T. & Shulman, G. I. Mechanisms for insulin resistance: Common threads and missing links. *Cell* **148**(5), 852–871. 10.1016/j.cell.2012.02.017 (2012). 22385956 10.1016/j.cell.2012.02.017 PMC3294420

[14] Kraus, F. *et al.* Function and regulation of the divisome for mitochondrial fission. *Nature* **590**(7844), 57–66. 10.1038/s41586-021-03214-x (2021). 33536648 10.1038/s41586-021-03214-x

[15] Liu, R. *et al.* Impaired mitochondrial dynamics and bioenergetics in diabetic skeletal muscle. *PLoS One* **9**(3), e92810. 10.1371/journal.pone.0092810 (2014). 24658162 10.1371/journal.pone.0092810 PMC3962456

[16] Sergi, D. *et al.* Mitochondrial (Dys)function and insulin resistance: From pathophysiological molecular mechanisms to the impact of diet. *Front. Physiol.* **10**, 532. 10.3389/fphys.2019.00532 (2019). 31130874 10.3389/fphys.2019.00532 PMC6510277

[17] Magkos, F., Hjorth, M. F. & Astrup, A. Diet and exercise in the prevention and treatment of type 2 diabetes mellitus. *Nat. Rev. Endocrinol.* **16**(10), 545–555. 10.1038/s41574-020-0381-5 (2020). 32690918 10.1038/s41574-020-0381-5

[18] Frodermann, V. *et al.* Exercise reduces inflammatory cell production and cardiovascular inflammation via instruction of hematopoietic progenitor cells. *Nat. Med.* **25**(11), 1761–1771. 10.1038/s41591-019-0633-x (2019). 31700184 10.1038/s41591-019-0633-x PMC6858591

[19] Wang, D. *et al.* Exercise-induced browning of white adipose tissue and improving skeletal muscle insulin sensitivity in obese/non-obese growing mice: Do not neglect exosomal miR-27a. *Front. Nutr.* **9**, 940673. 10.3389/fnut.2022.940673 (2022). 35782940 10.3389/fnut.2022.940673 PMC9248804

[20] Axelrod, C. L. *et al.* Exercise training remodels human skeletal muscle mitochondrial fission and fusion machinery towards a pro-elongation phenotype. *Acta Physiol. (Oxf.)* **225**(4), e13216. 10.1111/apha.13216 (2019). 30408342 10.1111/apha.13216 PMC6416060

[21] Zhao, R. Irisin at the crossroads of inter-organ communications: Challenge and implications. *Front. Endocrinol.*10.3389/fendo.2022.989135 (2022). 10.3389/fendo.2022.989135 PMC9578559 36267573

[22] Cariati, I. *et al.* Role of physical activity in bone-muscle crosstalk: Biological aspects and clinical implications. *J. Funct. Morphol. Kinesiol.*10.3390/jfmk6020055 (2021). 34205747 10.3390/jfmk6020055 PMC8293201

[23] Pedersen, B. K. Physical activity and muscle-brain crosstalk. *Nat. Rev. Endocrinol.* **15**(7), 383–392. 10.1038/s41574-019-0174-x (2019). 30837717 10.1038/s41574-019-0174-x

[24] Bostrom, P. *et al.* A PGC1-alpha-dependent myokine that drives brown-fat-like development of white fat and thermogenesis. *Nature* **481**(7382), 463–468. 10.1038/nature10777 (2012). 22237023 10.1038/nature10777 PMC3522098

[25] Bi, J. *et al.* Irisin alleviates liver ischemia-reperfusion injury by inhibiting excessive mitochondrial fission, promoting mitochondrial biogenesis and decreasing oxidative stress. *Redox Biol.* **20**, 296–306. 10.1016/j.redox.2018.10.019 (2019). 30388684 10.1016/j.redox.2018.10.019 PMC6216086

[26] Perakakis, N. *et al.* Physiology and role of irisin in glucose homeostasis. *Nat. Rev. Endocrinol.* **13**(6), 324–337. 10.1038/nrendo.2016.221 (2017). 28211512 10.1038/nrendo.2016.221 PMC5878942

[27] Mazur-Bialy, A. I., Pochec, E. & Zarawski, M. Anti-inflammatory properties of irisin, mediator of physical activity, are connected with TLR4/MyD88 signaling pathway activation. *Int. J. Mol. Sci.*10.3390/ijms18040701 (2017). 28346354 10.3390/ijms18040701 PMC5412287

[28] Toyama, E. Q. *et al.* Metabolism. AMP-activated protein kinase mediates mitochondrial fission in response to energy stress. *Science* **351**(6270), 275–281. 10.1126/science.aab4138 (2016). 26816379 10.1126/science.aab4138 PMC4852862

[29] Fan, J. *et al.* Protective effects of irisin on hypoxia-reoxygenation injury in hyperglycemia-treated cardiomyocytes: Role of AMPK pathway and mitochondrial protection. *J. Cell Physiol.* **235**(2), 1165–1174. 10.1002/jcp.29030 (2020). 31268170 10.1002/jcp.29030

[30] Kowluru, R. A. Retinopathy in a diet-induced type 2 diabetic rat model and role of epigenetic modifications. *Diabetes* **69**(4), 689–698. 10.2337/db19-1009 (2020). 31949005 10.2337/db19-1009 PMC7085254

[31] Kim, H. *et al.* Irisin mediates effects on bone and fat via alphaV integrin receptors. *Cell* **178**(2), 507–508. 10.1016/j.cell.2019.06.028 (2019). 31299203 10.1016/j.cell.2019.06.028 PMC6707723

[32] Bedford, T. G. *et al.* Maximum oxygen consumption of rats and its changes with various experimental procedures. *J. Appl. Physiol. Respir. Environ. Exerc. Physiol.* **47**(6), 1278–1283. 10.1152/jappl.1979.47.6.1278 (1979). 536299 10.1152/jappl.1979.47.6.1278

[33] Zhao, R. *et al.* Irisin regulating skeletal response to endurance exercise in ovariectomized mice by promoting Akt/beta-catenin pathway. *Front. Physiol.* **12**, 639066. 10.3389/fphys.2021.639066 (2021). 33841178 10.3389/fphys.2021.639066 PMC8027323

[34] Meyer, C. *et al.* Different mechanisms for impaired fasting glucose and impaired postprandial glucose tolerance in humans. *Diabetes Care* **29**(8), 1909–1914. 10.2337/dc06-0438 (2006). 16873801 10.2337/dc06-0438

[35] Wick, M. R. The hematoxylin and eosin stain in anatomic pathology-An often-neglected focus of quality assurance in the laboratory. *Semin. Diagn. Pathol.* **36**(5), 303–311. 10.1053/j.semdp.2019.06.003 (2019). 31230963 10.1053/j.semdp.2019.06.003

[36] Wu, H. & Ballantyne, C. M. Skeletal muscle inflammation and insulin resistance in obesity. *J. Clin. Investig.* **127**(1), 43–54. 10.1172/JCI88880 (2017). 28045398 10.1172/JCI88880 PMC5199705

[37] Bach, D. *et al.* Expression of Mfn2, the Charcot-Marie-Tooth neuropathy type 2A gene, in human skeletal muscle: Effects of type 2 diabetes, obesity, weight loss, and the regulatory role of tumor necrosis factor alpha and interleukin-6. *Diabetes* **54**(9), 2685–2693. 10.2337/diabetes.54.9.2685 (2005). 16123358 10.2337/diabetes.54.9.2685

[38] Lin, J. J. *et al.* Molecular basis of irisin regulating the effects of exercise on insulin resistance. *Appl. Sci.-Basel*10.3390/app12125837 (2022).36777332 10.3390/app12125837

[39] Zheng, S. *et al.* Irisin alleviates FFA induced beta-cell insulin resistance and inflammatory response through activating PI3K/AKT/FOXO1 signaling pathway. *Endocrine* **75**(3), 740–751. 10.1007/s12020-021-02875-y (2022). 34546489 10.1007/s12020-021-02875-y

[40] Wang, B. *et al.* Exercise ameliorating myocardial injury in type 2 diabetic rats by inhibiting excessive mitochondrial fission involving increased irisin expression and AMP-activated protein kinase phosphorylation. *J. Diabetes*10.1111/1753-0407.13475 (2023). 37721125 10.1111/1753-0407.13475 PMC10809304

[41] Lambert, K. *et al.* Combination of nutritional polyphenols supplementation with exercise training counteracts insulin resistance and improves endurance in high-fat diet-induced obese rats. *Sci. Rep.* **8**(1), 2885. 10.1038/s41598-018-21287-z (2018). 29440695 10.1038/s41598-018-21287-z PMC5811550

[42] Lu, Y. *et al.* Swimming exercise increases serum irisin level and reduces body fat mass in high-fat-diet fed Wistar rats. *Lipids Health Dis.* **15**, 93. 10.1186/s12944-016-0263-y (2016). 27177924 10.1186/s12944-016-0263-y PMC4866429

[43] Magalhaes, J. P. *et al.* Effectiveness of high-intensity interval training combined with resistance training versus continuous moderate-intensity training combined with resistance training in patients with type 2 diabetes: A one-year randomized controlled trial. *Diabetes Obes. Metab.* **21**(3), 550–559. 10.1111/dom.13551 (2019). 30284352 10.1111/dom.13551

[44] Luo, Y. *et al.* Disordered metabolism in mice lacking irisin. *Sci. Rep.* **10**(1), 17368. 10.1038/s41598-020-74588-7 (2020). 33060792 10.1038/s41598-020-74588-7 PMC7567109

[45] Mazur-Bialy, A. I. *et al.* New insight into the direct anti-inflammatory activity of a myokine irisin against proinflammatory activation of adipocytes. Implication for exercise in obesity. *J. Physiol. Pharmacol.* **68**(2), 243–251 (2017). 28614774

[46] Irazoki, A. *et al.* Coordination of mitochondrial and lysosomal homeostasis mitigates inflammation and muscle atrophy during aging. *Aging Cell*10.1111/acel.13583 (2022). 35263007 10.1111/acel.13583 PMC9009131

[47] Hood, D. A. *et al.* Maintenance of skeletal muscle mitochondria in health, exercise, and aging. *Annu. Rev. Physiol.* **81**, 19–41. 10.1146/annurev-physiol-020518-114310 (2019). 30216742 10.1146/annurev-physiol-020518-114310

[48] Fealy, C. E. *et al.* Exercise training decreases activation of the mitochondrial fission protein dynamin-related protein-1 in insulin-resistant human skeletal muscle. *J. Appl. Physiol. (1985)* **117**(3), 239–245. 10.1152/japplphysiol.01064.2013 (2014). 24947026 10.1152/japplphysiol.01064.2013 PMC4122691

[49] Cunningham, J. T. *et al.* mTOR controls mitochondrial oxidative function through a YY1-PGC-1alpha transcriptional complex. *Nature* **450**(7170), 736–740. 10.1038/nature06322 (2007). 18046414 10.1038/nature06322

[50] Zhang, X. *et al.* Irisin exhibits neuroprotection by preventing mitochondrial damage in Parkinson’s disease. *NPJ Parkinsons Dis.* **9**(1), 13. 10.1038/s41531-023-00453-9 (2023). 36720890 10.1038/s41531-023-00453-9 PMC9889817

[51] Chen, N. *et al.* Irisin, an exercise-induced myokine as a metabolic regulator: An updated narrative review. *Diabetes Metab. Res. Rev.* **32**(1), 51–59. 10.1002/dmrr.2660 (2016). 25952527 10.1002/dmrr.2660

[52] Islam, M. R. *et al.* Exercise hormone irisin is a critical regulator of cognitive function. *Nat. Metab.* **3**(8), 1058–1070. 10.1038/s42255-021-00438-z (2021). 34417591 10.1038/s42255-021-00438-z PMC10317538

[53] Lee, H. J. *et al.* Irisin, a novel myokine, regulates glucose uptake in skeletal muscle cells via AMPK. *Mol. Endocrinol.* **29**(6), 873–881. 10.1210/me.2014-1353 (2015). 25826445 10.1210/me.2014-1353 PMC5414737

[54] Tang, H. *et al.* Irisin inhibits hepatic cholesterol synthesis via AMPK-SREBP2 signaling. *EBioMedicine* **6**, 139–148. 10.1016/j.ebiom.2016.02.041 (2016). 27211556 10.1016/j.ebiom.2016.02.041 PMC4856751

[55] Hoffman, N. J. *et al.* Global phosphoproteomic analysis of human skeletal muscle reveals a network of exercise-regulated kinases and AMPK substrates. *Cell Metab.* **22**(5), 922–935. 10.1016/j.cmet.2015.09.001 (2015). 26437602 10.1016/j.cmet.2015.09.001 PMC4635038

[56] Kim, H. *et al.* Fine-tuning of Drp1/Fis1 availability by AKAP121/Siah2 regulates mitochondrial adaptation to hypoxia. *Mol. Cell* **44**(4), 532–544. 10.1016/j.molcel.2011.08.045 (2011). 22099302 10.1016/j.molcel.2011.08.045 PMC3360955

