# Author Correction: Exercise ameliorates muscular excessive mitochondrial fission, insulin resistance and inflammation in diabetic rats via irisin/AMPK activation

**DOI:** 10.1038/s41598-024-70170-7

**Published:** 2024-08-19

**Authors:** Junjie Lin, Xin Zhang, Yu Sun, Haocheng Xu, Nan Li, Yuanxin Wang, Xin Tian, Chen Zhao, Bin Wang, Baishu Zhu, Renqing Zhao

**Affiliations:** College of Physical Education, Yangzhou University, Yangzhou, 225009 China

Correction to: *Scientifc Reports* 10.1038/s41598-024-61415-6, published online 09 May 2024

The original version of this Article contained an error in the Funding section.

“This work was supported by the Natural Science Foundation of Jiangsu Province (BK20201435) and Postgraduate Research & Practice Innovation Program of Jiangsu Province (3733).”

now reads:

“This work was supported by the Natural Science Foundation of Jiangsu Province (BK20201435) and Postgraduate Research & Practice Innovation Program of Jiangsu Province (KYCX24_3730).”

The original Article has been corrected.

