# Supplementary material for: Exercise ameliorates muscular excessive mitochondrial fission, insulin resistance and inflammation in diabetic rats via irisin/AMPK activation
Source: Sci Rep. 2024 May 9;14:10658. doi: 10.1038/s41598-024-61415-6 (PMC11082241; doi:10.1038/s41598-024-61415-6)
Supplement: Supplementary file 1 — Supplementary Figures. [file 41598_2024_61415_MOESM1_ESM.pdf]

Figure S1

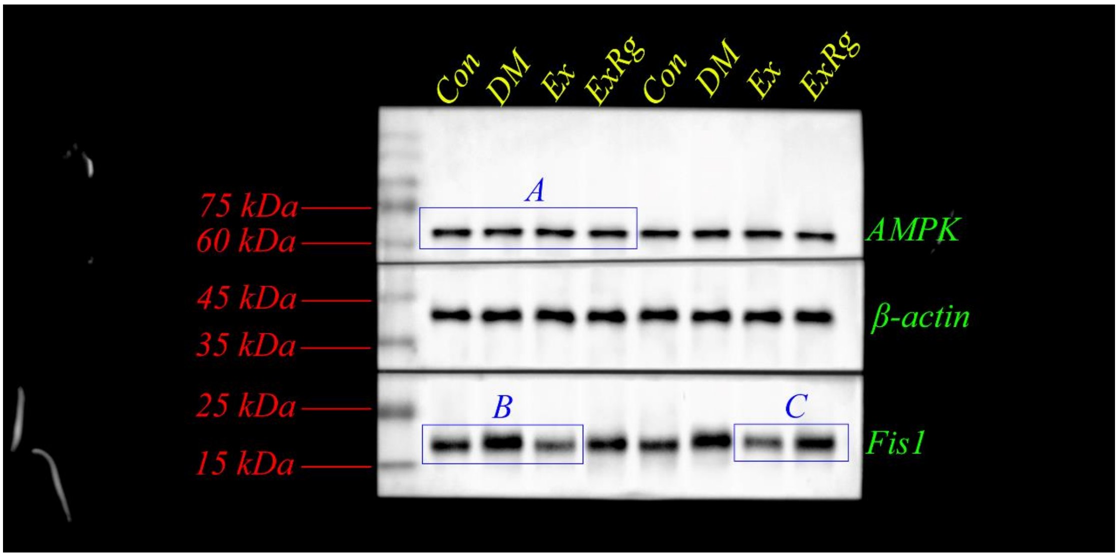

(A) AMPK protein bands in Figure 5B.

(B) Fis1 protein bands in Figure 4E.

(C) Fis1 protein bands in Figure 7E.

Figure S2

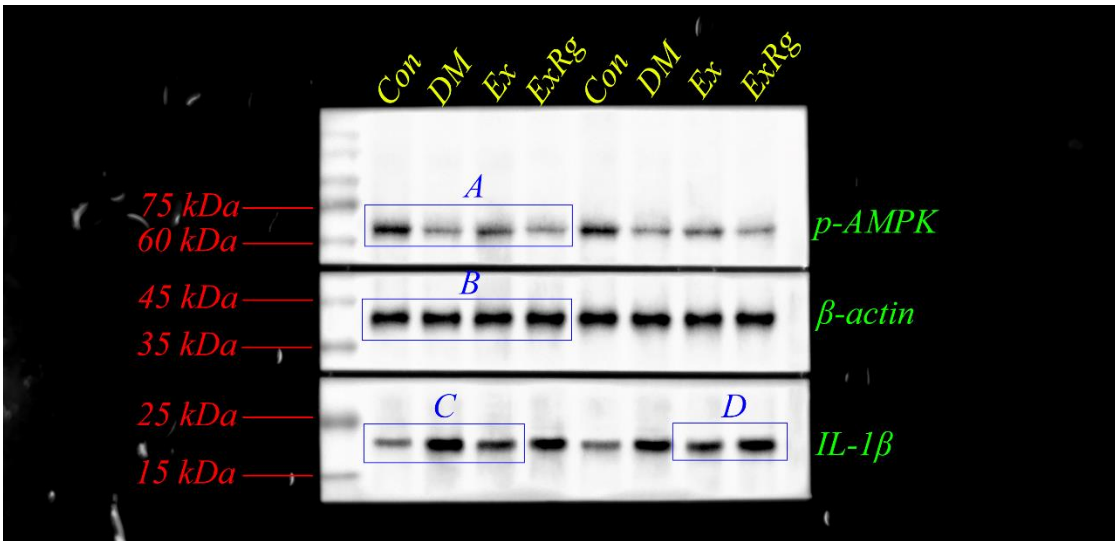

(A) p-AMPK protein bands in Figure 5B.

(B) β-actin protein bands in Figure 5B.

(C) IL-1β protein bands in Figure 3F.

(D) IL-1β protein bands in Figure 6P.

Figure S3

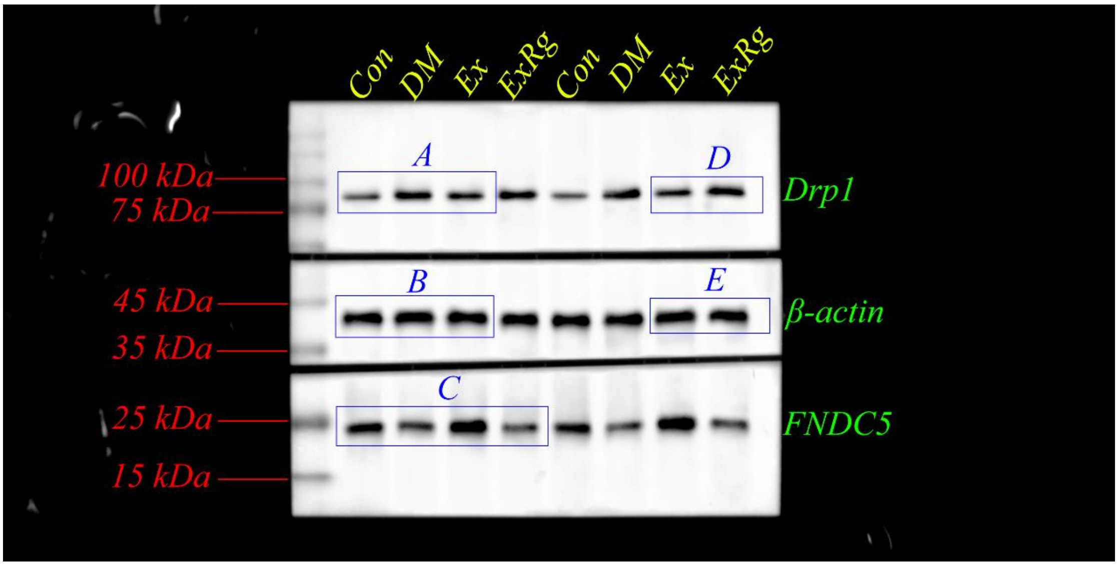

(A) Drp1 protein bands in Figure 4E.

(B) β-actin protein bands in Figure 4E.

(C) FNDC5 protein bands in Figure 5B.

(D) Drp1 protein bands in Figure 7E.

(E) β-actin protein bands in Figure 7E.

Figure S4

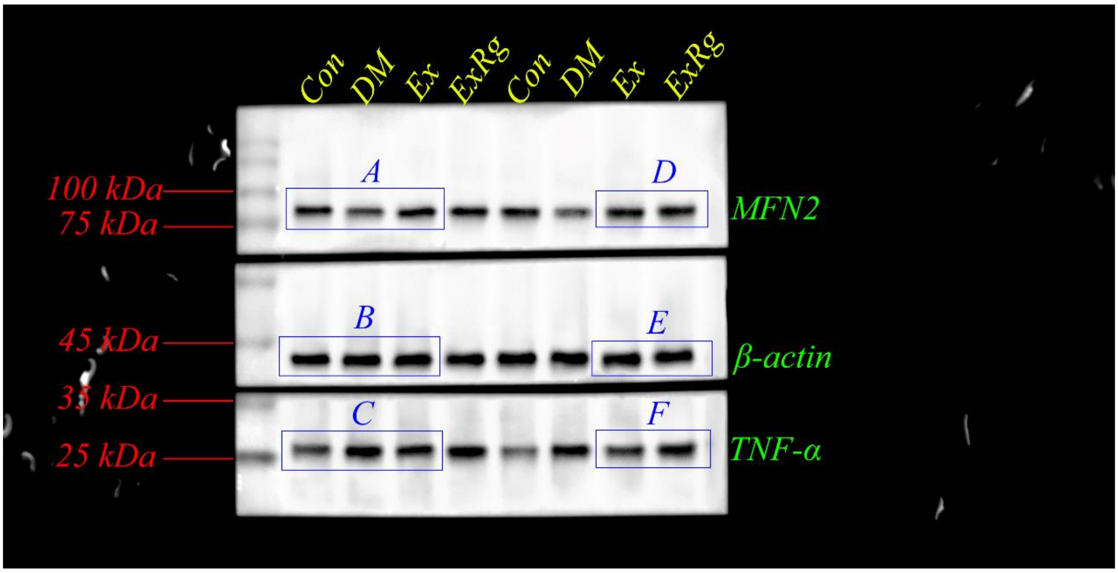

(A) MFN2 protein bands in Figure 4E.

(B) β-actin protein bands in Figure 3F.

(C) TNF-α protein bands in Figure 3F.

(D) MFN2 protein bands in Figure 7E.

(E) β-actin protein bands in Figure 6P.

(F) TNF-α protein bands in Figure 6P.

Figure S5

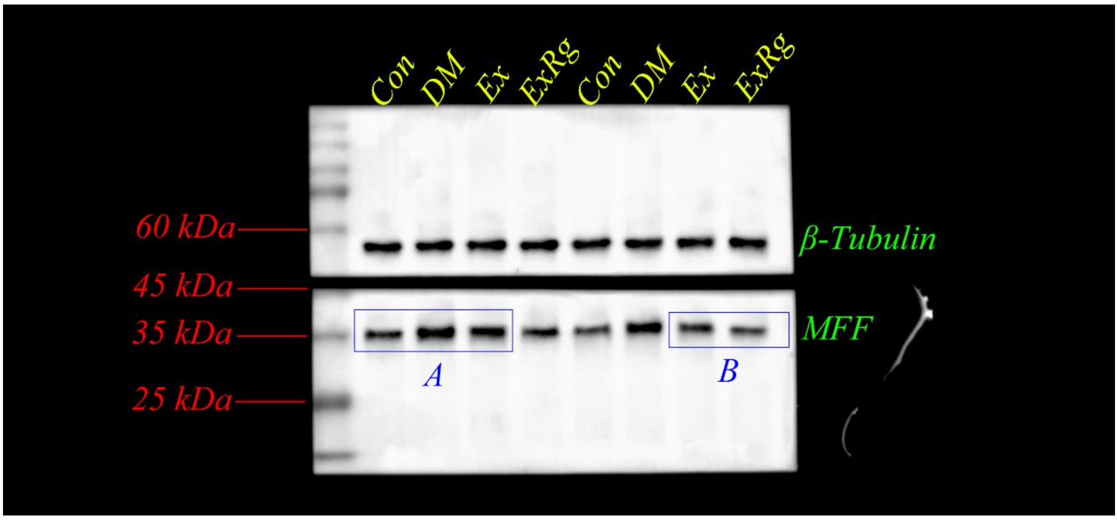

(A) MFF protein bands in Figure 4E.

(B) MFF protein bands in Figure 7E.
